# Core symptoms in the cardiac rehabilitation phase of patients with chronic heart failure: A symptom network analysis study

**DOI:** 10.1097/MD.0000000000045204

**Published:** 2025-10-17

**Authors:** Fengfei Zhou, Bailing Zhang, Yi Lu, Qingqing Yang

**Affiliations:** a Department of Cardiology, The Sixth People’s Hospital of Nantong, Nantong, Jiangsu, China.

**Keywords:** cardiac rehabilitation, heart failure, network analysis, symptoms

## Abstract

This study investigates the composition characteristics of symptom clusters in patients with chronic heart failure (CHF) during the cardiac rehabilitation phase, constructs their symptom network structure, and identifies core symptoms, thereby providing a foundation for optimizing the precise management plan for symptoms during the rehabilitation period. A convenience sampling method was employed. From January 2023 to May 2025, 550 patients with CHF undergoing cardiac rehabilitation at the Department of Cardiology of Nantong Sixth People’s Hospital were selected for a cross-sectional survey. The patients’ symptom experiences were assessed using a general information questionnaire and the Chinese version of the Memorial Heart Failure Symptom Assessment Scale for Heart Failure. The analysis primarily focused on the degree of symptom distress, and exploratory factor analysis was conducted to extract symptom clusters. The R 4.3.1 software was utilized to construct a symptom network model, and node strength and closeness centrality were calculated to identify core symptoms and their interrelationships. The identification of symptom clusters through exploratory factor analysis revealed 5 distinct clusters that accounted for 63.75% of the variance. These clusters include: cardiopulmonary function limitation, fluid retention imbalance, fatigue-nutritional disorder, gastrointestinal symptoms, and neuropsychological disorders. Notably, shortness of breath following activity (r_s = 4.618), fatigue (r_s = 4.752), and anxiety (r_s = 4.752) emerged as the core hubs of the network, with fatigue exhibiting a predictability of up to 69%. Medical staff should prioritize intervening in the 3 core symptoms, paying attention to their cross-group correlations, and implementing precise management strategies based on the symptom network structure to effectively reduce the symptom burden during the recovery period, promote functional recovery, and improve the quality of life.

## 1. Introduction

Chronic heart failure (CHF) is a clinical syndrome caused by myocardial injury, abnormal load, or dysfunction in ventricular filling and ejection, characterized by typical clinical features such as dyspnea, fluid retention, and significant decline in exercise tolerance.^[1]^ According to the “China Cardiovascular Health and Disease Report 2023,”^[2]^ the number of CHF patients in China has reached 8.9 million, with 10.29 million hospitalizations for HF in 2022, and the 30-day readmission rate for patients was 10.0%, imposing a heavy burden on social medical resources. Currently, guideline-directed medical therapy, cardiac rehabilitation (CR), and multidisciplinary collaboration form the core treatment system for CHF.^[3]^

However, due to the dual impact of the complex pathophysiological mechanisms of the disease and the side effects of treatment, CHF patients often experience multiple symptoms during the rehabilitation stage. These symptoms include exertional dyspnea, persistent fatigue, peripheral edema, sleep disorders, anxiety, depression, and cognitive decline, among others.^[4,5]^ These symptoms do not exist in isolation but are intertwined and mutually reinforcing, forming a dynamic “symptom cluster.”^[6]^ As a stable cluster composed of 2 or more symptoms, the interaction within the symptom cluster accelerates the functional decline of patients and significantly worsens their prognosis.

In previous studies, latent variable models such as factor analysis were primarily used to identify symptom clusters. These methods are based on the assumption that symptoms are influenced by underlying common mechanisms,^[7]^ yet they have limitations in uncovering the direct dynamic relationships between symptoms. In contrast, network analysis quantifies the “centrality” of each symptom by constructing a symptom interaction model, accurately identifies core symptoms that require priority intervention, and analyzes the potential regulatory pathways of symptom clusters.^[8]^ For instance, fatigue can indirectly worsen edema by decreasing the patient’s exercise compliance, while anxiety may intensify the patient’s subjective experience of dyspnea.^[9]^ Currently, network analysis has been applied to the symptom research of cancer^[10]^ and stroke patients,^[11]^ but the symptom cluster research based on this method remains unexplored in the field of CR for CHF. This study aims to construct a symptom network of CHF patients during the CR phase through a cross-sectional survey, identify core symptoms and interaction patterns within symptom clusters, and provide targets for cross-symptom joint intervention, thereby optimizing rehabilitation management strategies and enhancing the quality of life and long-term prognosis of patients.

## 2. Study subjects and methods

### 2.1. Subjects

Patients hospitalized and undergoing CR in the Department of Cardiology at the Sixth People’s Hospital of Nantong between January 2023 and May 2025 were selected using convenience sampling. Inclusion criteria were as follows: Meeting the diagnostic criteria for CHF as outlined in the “Chinese Guidelines for the Diagnosis and Treatment of Heart Failure”^[12]^; Being in the stable phase of CR (New York Heart Association (NYHA) class II–III) and having participated regularly in an outpatient or home CR program for over 4 weeks; Being at least 18 years old; Being conscious, capable of communicating and understanding independently, and able to voluntarily sign informed consent. Exclusion criteria included: Secondary heart failure, such as hyperthyroid cardiomyopathy, alcoholic cardiomyopathy, or those with other end-stage diseases; Suffering from severe mental illness or cognitive impairment (with a Mini-Mental State Examination (MMSE) score < 24); Having contraindications to exercise rehabilitation, such as unstable angina or uncontrolled arrhythmia.

### 2.2. Sample size calculation

The Chinese version of the Memorial Heart Failure Symptom Assessment Scale, which includes 32 symptom items, was utilized in this study. Following the principle of the regularized network model (EBICglasso) proposed by Epskamp et al,^[13]^ the sample size should include at least 15 cases for each variable (i.e., 32 variables × 15 cases = 480 cases), and the theoretical sample size should be 576 cases after accounting for a 20% nonresponse rate. This study has been reviewed and approved by the Ethics Committee of the Sixth People’s Hospital of Nantong, with the ethics approval number being (NTLYLL2023015).

### 2.3. Instrument of survey

#### 2.3.1. General information questionnaire

After consulting relevant literature on heart failure and integrating clinical practice, researchers independently compiled a general information questionnaire for heart failure patients. The content is divided into 2 parts: (1) Demographic data, which includes age, gender, BMI, smoking history, drinking history, occupation type, and educational level; and (2) Disease-related data, which encompasses cardiac function classification, left ventricular ejection fraction, NT-proBNP level, complications such as hypertension, diabetes, chronic kidney disease, and types of medication.

#### 2.3.2. Chinese version of the Memorial Heart Failure Symptom Assessment Scale

This scale was developed by Zambroski et al^[14]^ and subsequently translated into Chinese and validated in China by Guo Jinyu team.^[15]^ The Chinese version comprises 32 symptom items across 3 dimensions: physical symptoms (21 items related to cardiovascular, respiratory, digestive systems, etc); psychological symptoms (6 items, including anxiety and depression); and heart failure-specific symptoms (5 items, such as orthopnea and decreased exercise tolerance). Each symptom item is assessed on 4 attributes: existence (yes or no); frequency, rated on a scale from 1 to 4, corresponding to rarely to almost constantly; severity, also rated on a scale from 1 to 4, corresponding to mild to very severe; and distress, scored on a scale from 0 to 4, corresponding to no distress to very severe distress. The symptom burden score is calculated by summing the scores of each symptom across the 3 dimensions of frequency, severity, and distress, and then dividing by the number of dimensions (3). The formula is as follows: Symptom burden score = (frequency score + severity score + distress score)/ 3. The Cronbach α coefficient of the scale in this study was 0.854, indicating good reliability and validity.

#### 2.3.3. Data collection methods

The data collection for this study was conducted by 2 supervisor nurses from the cardiology department who had undergone uniform training by the investigators. The study adhered strictly to a standardized data collection process. Prior to data collection, the purpose and significance of the study, along with the method for completing the questionnaire, were explained in detail to the patients and their families. Written informed consent was obtained after ensuring they were fully informed. Uniform instructions were provided to explain each item on the questionnaire. Patients capable of independently completing the questionnaire did so without assistance. For those unable to fill out the questionnaire due to health reasons, the data collector verbally went through each item with them and accurately recorded their subjective responses. The collector checked the completeness of each questionnaire on-site to ensure the accuracy of the data collected. This study deemed any questionnaire with 20% or more missing items as invalid. Throughout the study period, a total of 580 questionnaires were distributed, and 550 valid questionnaires were ultimately returned, yielding an effective recovery rate of 94.87%. Additionally, 14 patients declined to continue participating due to the questionnaire’s cumbersome content, and another 16 questionnaires were invalidated because the proportion of missing key items, such as core symptom scores, exceeded 50%.

### 2.4. statistical analysis

A rigorous data management and analysis strategy was employed. Initially, Excel 2019 software was utilized for double data entry verification to ensure the accuracy and consistency of data entry. SPSS 26.0 (IBM Corp., Armonk) software was then used for statistical analysis of demographic data. Measurement data were expressed as mean ± standard deviation or median (interquartile range), depending on the distribution characteristics of the data. Count data were described as frequency and percentage. For the data processing of symptom distress scores, exploratory factor analysis was applied for in-depth analysis. The specific operational steps were as follows: principal component analysis combined with the maximum variance rotation method was used to extract symptom factors, and the screening criteria were set as follows: the characteristic root threshold was >1.0; each factor included at least 2 symptom items. The loading value of symptom items on the factor was not <0.40. Symptom items with cross-loading were classified according to the highest loading value on each factor. Network model building and analysis based on R 4.3.1 software were completed, with the specific process including: network construction: calling the qgraph package to build an EBICglasso regularization network based on the partial correlation matrix of symptom distress, using the degree of partial correlation coefficient matrix as the input matrix. Spurious associations were effectively constrained by setting the penalty parameter γ of the minimum absolute shrinkage and variable selection model to 0.5. In the network, nodes represent different symptoms, the blue solid line edge represents a positive correlation between symptoms, the red dashed line edge represents a negative correlation, and the width of the edge intuitively reflects the correlation strength. Additionally, the Fruchterman-Reingold force-directed algorithm was used to optimize the node layout to enhance the network visualization effect. Node properties analysis: with the help of computing nodes predictability, MGM, and visualization with circular ribbon width. The identification of core symptoms was conducted based on central indicators such as intensity, closeness, mediation, and expected impact. Among these, the intensity index was the primary choice as the core evaluation basis, as it has been confirmed to have the best stability in numerous studies. Network stability validation: by performing 1000 Bootstrap sampling calculations to determine the stability of a 95% confidence interval, and calculating the centrality stability coefficient of the CS network stability assessment. The criteria are as follows: when CS > 0.25, the network stability is acceptable; when CS > 0.5, the network stability is good.

## 3. Result

### 3.1. General information of the survey subjects

A total of 550 patients with CHF undergoing CR were included in the study. Of these, 358 were male and 192 were female; their ages ranged from 53 to 86 years, with an average age of 68.24 ± 6.71 years. The details are presented in Table 1.

**Table 1:** General characteristics of the survey subjects (n = 550).

Project	Number	Percentage (%)
Gender		
Male	358	65.1
Female	192	34.9
BMI(kg/m²)		
<18.5	33	6.0
18.5~24.9	418	76.0
≥25	99	18.0
Educational attainment		
Primary school and below	145	26.4
junior high school	256	46.5
High school/technical secondary school	118	21.5
University and above	31	5.6
Status of occupation		
On the job	23	4.1
Retirement	381	69.3
Farming	146	26.6
History of smoking		
Yes	155	28.2
No	395	71.8
Alcohol consumption history		
Yes	191	34.7
No	359	65.3
Cardiac Function Classification		
NYHA class Ⅱ	248	45.1
NYHA class Ⅲ	302	54.9
Left ventricular ejection fraction		
HFrEF(LVEF ≤ 40%)	287	287
HFmrEF(40%<LVEF < 50%)	86	15.6
HFpEF(LVEF ≥ 50%)	177	32.2
NT-proBNP(pg/mL)		
≤900	92	16.7
>900	458	83.3
Comorbidity		
hypertension	425	77.3
Type 2 Diabetes Mellitus	205	37.3
Chronic kidney Disease	112	20.4
Chronic obstructive pulmonary disease	87	15.8
Course of heart failure (years)		
≤1	88	16.0
>1	462	84.0
Classes of Drugs		
ARNI/ACEI/ARB	514	93.5
beta-blockers	505	91.8
SGLT2i	309	56.2
MRA	271	49.3

### 3.2. Symptom occurrence and symptom cluster extraction results during CR in patients with CHF

This study conducted a factor analysis on 32 symptoms. The results of the prerequisite tests indicated that the KMO value was 0.812, and the Bartlett sphericity test yielded a χ^2^ of 1856.327 (*P* < .001), suggesting a strong correlation among the variables and satisfying the applicability criteria for factor analysis. Utilizing principal component analysis in conjunction with the maximum variance rotation method, 5 symptom clusters were ultimately identified, with a cumulative variance explanation rate of 63.75%, effectively uncovering the underlying structure among the symptoms. These 5 symptom clusters were named as follows: Cardiopulmonary Function Limitation Cluster (Factor 1), Fluid Retention Imbalance Cluster (Factor 2), Fatigue – Nutritional Imbalance Cluster (Factor 3), Gastrointestinal Symptom Cluster (Factor 4), and Neuropsychological Disorder Cluster (Factor 5). The loading values of the items within each symptom cluster on the corresponding factor were all >0.40, and no significant cross-loading was observed, suggesting that the extracted symptom cluster structure was stable and the division was justified. The specific composition of the symptom clusters and the factor loadings are detailed in Table 2.

**Table 2:** Symptom burden and symptom duration extraction results for cardiac rehabilitation stages in CHF (N = 550).

Project	Number of cases (%)	Symptom burden[points, M(P25, P75)]	Factor loading				
			Limited cardiopulmonary function group	Fluid retention imbalance group	Fatigue-nutritional Disorders cluster	Gastrointestinal symptom clusters	Neuropsychological disorders group
Shortness of breath after activity	488 (88.73)	2.67 (2.33, 3.00)	0.853	0.102	0.076	−0.094	0.115
Difficulty breathing when lying flat	376 (68.36)	2.33 (2.00, 2.67)	0.798	0.153	0.118	0.094	−0.063
Palpitations of heart	339 (61.64)	2.00 (1.67, 2.33)	0.722	0.095	0.086	0.122	0.064
Edema of the lower limbs	418 (76.00)	2.33 (2.00, 2.67)	0.187	0.817	0.033	0.105	−0.082
Weight gain	297 (54.00)	1.67 (1.33, 2.00)	0.094	0.793	0.061	0.217	−0.042
Fatigue	390 (70.91)	2.67 (2.00, 3.00)	0.166	0.041	0.792	−0.042	0.231
Loss of appetite	274 (49.82)	2.00 (1.33, 2.67)	0.153	0.028	0.841	0.175	−0.091
Abdominal distention	241 (43.82)	1.67 (1.33, 2.00)	−0.073	0.206	0.125	0.782	0.032
Nausea	194 (35.27)	1.33 (0.67, 2.00)	0.078	0.125	0.038	0.734	0.047
Feeling of anxiety	358 (65.09)	2.00 (1.67, 2.67)	−0.035	−0.177	0.201	0.038	0.828
Sleep disorders	238 (43.27)	2.00 (1.67, 2.67)	0.101	−0.055	0.154	0.013	0.769
Factor characteristic root	–	–	4.217	2.844	2.102	1.538	1.312
Cumulative variance contribution rate (%)	–	–	24.17	39.53	49.77	58.05	63.75

### 3.3. Network analysis of symptom clusters in patients with CHF

**Figure 1.**
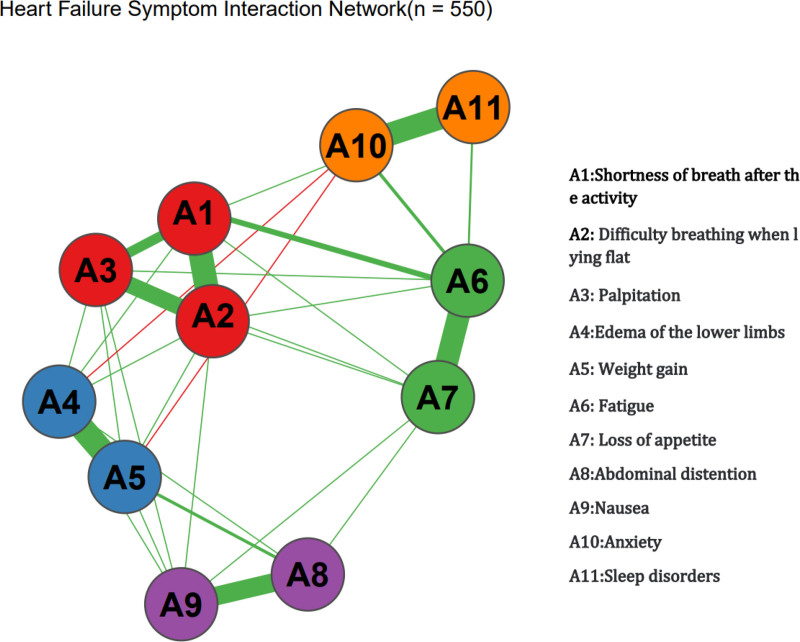
Core symptom network diagram of heart failure patients during cardiac rehabilitation. The figure displays the ranking of symptoms based on 3 centrality indices from the network analysis: Strength (the total sum of connection weights), betweenness (the frequency of lying on the shortest path between other symptoms), and closeness (the average distance to all other symptoms). Symptoms A1 (dyspnea on exertion), A6 (fatigue), and A10 (anxiety) consistently exhibited the highest values across all 3 measures, identifying them as the most influential core symptoms within the network. These central symptoms are prime targets for prioritized clinical intervention.

**Figure 2.**
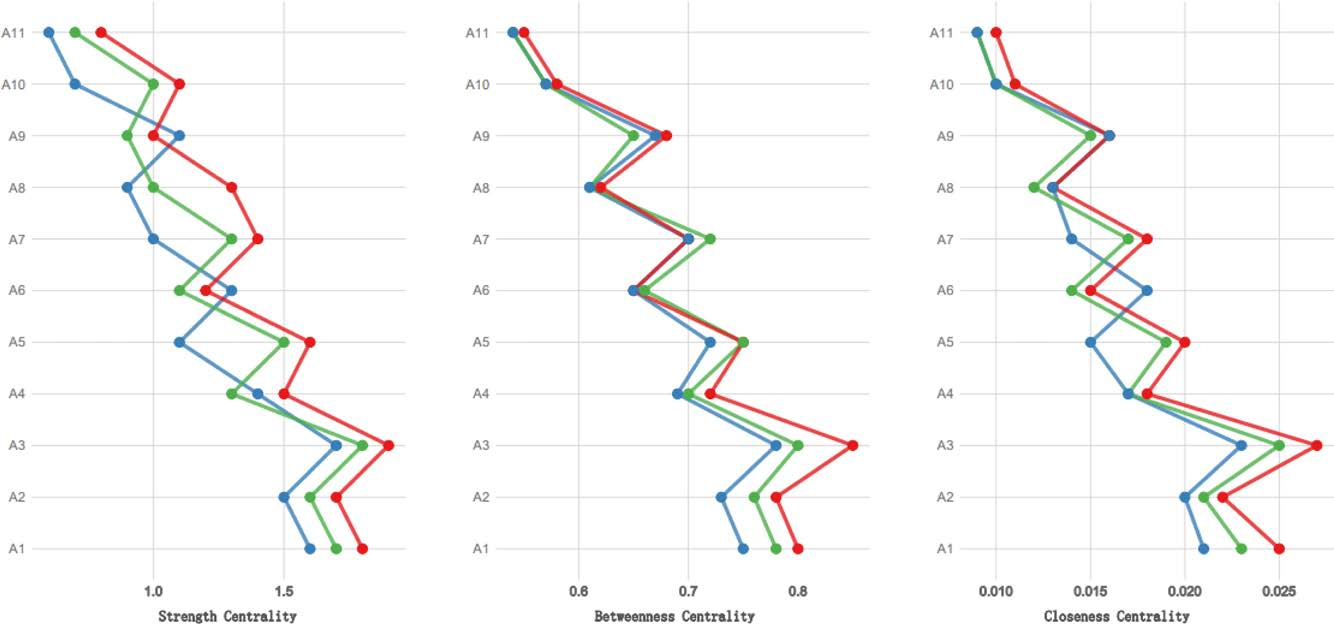
Symptom network centrality indicators during cardiac rehabilitation in patients with CHF. This plot assesses the accuracy and stability of the estimated edge weights (symptom–symptom associations) through nonparametric bootstrapping (1000 samples). Each horizontal line represents one edge in the network. The red dots indicate the edge weight estimated from the original sample. The black dots represent the mean edge weight across all bootstrap samples. The horizontal lines depict the 95% confidence intervals (CI). Narrow CIs (e.g., edges at the top of the plot, such as those involving core symptoms A1, A6, and A10) indicate precise and stable estimates, meaning these robust connections are unlikely to be false positives. Wider CIs suggest less stable edges that should be interpreted with caution.

**Figure 3.**
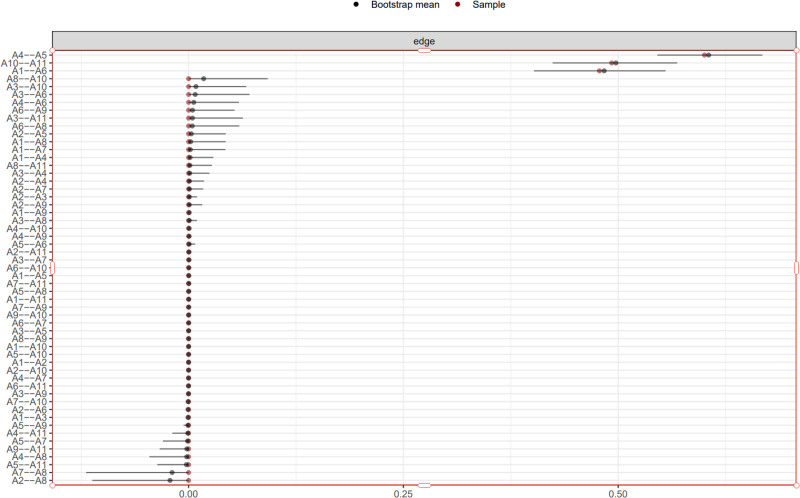
Bootstrap confidence intervals for edge weights in symptom network.

In the symptom network model, the thickness and color depth of the edges visually represent the strength of the correlations between symptoms. As depicted in Figure 1, “Shortness of breath after activity” (A1), “Fatigue” (A6), and “Anxiety” (A10) constitute the central hubs of the network, with the thickest and darkest edges connecting these 3 nodes (partial correlation coefficients *R* = 0.76–0.85), indicating a significant symptom synergy effect. The predictability of the nodes is depicted in concentric circles (range: 18%–69%), with “A6 Fatigue” exhibiting the highest predictability at 69%, suggesting that 69% of the variation in this symptom can be explained by neighboring symptoms; “A1 Shortness of breath after activity” has a predictability of 65%, reflecting its strong association with “Palpitations of heart” (A3) and “Difficulty breathing when lying flat” (A2). The reliability of the network was assessed through 1000 Bootstrap resamplings, with the outcomes indicating that the strength CS coefficient was 0.781 (95% confidence intervals (CI): 0.743–0.819), the density CS coefficient was 0.692 (95% CI: 0.652–0.732), and the centrality CS coefficient was 0.538 (95% CI: 0.502–0.574), all CS coefficients exceeding 0.5. The confidence interval width of the centrality indicators for the core symptoms (A1, A6, A10) was <0.05, confirming that the network structure possesses good stability and the accuracy of the centrality indicators’ estimation is high. Detailed data are presented in Table 3, and the visualization results of the network structure and node attributes are illustrated in Figures 2 and 3.

**Table 3:** Core symptom identification.

•Symptom	Strength centrality (rs)	Closeness centrality (rc)	Betweenness centrality (rb)
Fatigue (A6)	4.752	0.0143	0.028
Shortness of breath after exercise (A1)	4.618	0.0138	0.025
Anxiety (A10)	4.302	0.0129	0.021

## 4. Discussion

This study innovatively employed symptom network analysis to systematically examine the characteristics and interaction mechanisms of core symptoms in patients with CHF undergoing CR. The research confirmed that shortness of breath (88.73%), fatigue (70.91%), and anxiety (65.09%) were the central hubs of the symptom network. These symptoms exhibited high intensity values (r_s > 4.3) and high predictability (>65%), indicating that they are key drivers influencing the rehabilitation process. This finding aligns closely with the pathophysiological mechanism of CHF: reduced cardiac output results in tissue hypoperfusion, which causes skeletal muscle metabolic disorders and mitochondrial dysfunction, directly leading to fatigue.^[16]^ Elevated left ventricular filling pressure results in pulmonary venous hypertension, accompanied by pulmonary interstitial edema, ultimately manifesting as exercise-induced shortness of breath.^[17]^ The persistent activation of the sympathetic nervous system promotes the excessive release of norepinephrine, which leads to hyperactivity of the amygdala, and subsequently produces anxiety.^[18]^

Among the symptoms, fatigue exhibits the highest intensity within the symptom network (rs = 4.752), and it, along with anorexia, constitutes the “fatigue-malnutrition cluster.” The mechanism of its formation involves 3 levels: First, cardiac dysfunction and energy metabolism disorders,^[19]^ where reduced cardiac output results in insufficient tissue perfusion and decreased blood flow to skeletal muscles, leading to a decline in mitochondrial oxidative phosphorylation efficiency. This blocks ATP synthesis and the accumulation of muscle lactic acid activates type III/IV afferent nerve fibers, which, through the central inhibitory pathway, exacerbates fatigue. Second, the activation of the neuroendocrine-inflammatory axis.^[20]^ In the state of CHF, the sympathetic nervous system and the hypothalamic-pituitary-adrenal axis (HPA axis) are overly activated, and a large amount of pro-inflammatory cytokines (such as TNF-α, IL-6) are released. These not only inhibit the hypothalamic feeding center but also interfere with the central fatigue regulation mechanism through the blood-brain barrier, creating a “fatigue-malnutrition” vicious cycle. Third, during CR training, the sudden increase in ventilation demand leads to a significant rise in oxygen consumption by respiratory muscles, further exacerbating the imbalance between energy supply and demand throughout the body. This energy distribution crisis makes fatigue the primary restrictive symptom that hinders exercise rehabilitation.

The results of this study indicate that exertional shortness of breath, with an intensity value of r_s = 4.618, is strongly correlated with palpitations (A3) and dyspnea upon lying down (A2). This phenomenon is closely linked to hemodynamic disorders. Furthermore, the imbalance in pulmonary vascular hydrostatic pressure resulting from changes in posture can lead to positional dyspnea. Particularly during CR training, if a patient’s heart rate rises by more than 35 beats per minute postexercise, it suggests that the cardiopulmonary load has reached or surpassed the compensatory threshold. At this juncture, it is imperative to promptly adjust the exercise prescription to prevent the induction of acute decompensation. Concurrently, research has established a stable and robust correlation between lower limb edema and weight gain, aligning with the neuroendocrine activation mechanism of heart failure.^[21]^ When the renin-angiotensin-aldosterone system is abnormally activated, the body experiences water and sodium retention, resulting in fluid overload. Clinically, this is manifested as rapid weight gain and peripheral tissue edema. Additionally, the significant correlation between anxiety and sleep disorders uncovers a “psychophysiological bidirectional pathway” in patients with heart failure. On one hand, symptoms of heart failure, such as paroxysmal nocturnal dyspnea, can trigger anxiety; on the other hand, anxiety and sleep disorders perpetuate a vicious cycle by activating the sympathetic nervous system and stimulating neuroendocrine responses,^[22]^ which further exacerbates the pathological process of heart failure. Based on these findings, multi-dimensional intervention strategies are recommended in CR programs. For volume management, sodium restriction programs and the rational use of diuretics should be tailored to individual patient differences. At the psychological intervention level, cognitive behavioral therapy can be introduced to enhance anxiety and sleep quality, and to break the cycle of “psychophysiological” interaction deterioration, thereby improving the rehabilitation outcome.

It is noteworthy that the predictability of fatigue is as high as 69.1%, suggesting that nearly 70% of symptom variations can be accounted for by changes in neighboring symptoms. This provides a theoretical foundation for targeted interventions. According to the results of network centrality analysis, fatigue emerges as the primary intervention target, with an intensity value of r_s = 4.752 and a moderate value of r_b = 0.028. Seifi et al^[23]^ discovered that 1 hour of daily muscle relaxation training, coupled with natural sound therapy, can significantly alleviate fatigue symptoms in patients with CHF. Wang et al^[24]^ created a personalized fatigue management plan, tailored to individual needs, social support, and lifestyle, through personalized fatigue management counseling. This led to a significant reduction in fatigue levels among participants after 12 weeks. Furthermore, the stepwise fatigue management strategy recommended by the American Heart Association (AHA) guidelines is worth considering^[25]^: initially, exercise tolerance is evaluated using the 6-minute walk test (6MWT), and for patients with a 6MWT < 300m, endurance training at 40% of VO2 peak intensity is conducted daily for 20 minutes; in the second phase, omega-3 fatty acids (2g/d) and high-density lipoprotein (1.2g/kg/d) are supplemented; in the final phase, remote monitoring and optimized management are initiated for patients with NT-proBNP > 900pg/mL.^[26]^ It is advised that during CR, patients be guided to perform diaphragmatic breathing exercises 3 times daily and utilize blood flow-directed inspiratory capacity measurement to alleviate respiratory symptoms.^[27]^ The management of psychological symptom clusters necessitates the integration of both physiological and psychological intervention pathways. The physiological pathway involves heart rate variability biofeedback to stabilize the LF/HF ratio within the range of 1.5–2.0^[28]^; the psychological intervention concentrates on cognitive behavioral therapy to correct catastrophic thinking about the disease and incorporates mindfulness-based stress reduction training.^[29,30]^ For patients with a Hospital Anxiety and Depression Scale (HADS) score > 11, escitalopram (10mg/d) can serve as a short-term transitional treatment within the physiological–psychological intervention framework.^[31]^

This study has the following limitations at the methodological and clinical levels: Despite the exclusion of secondary heart failure (e.g., hypertrophic cardiomyopathy), the included heart failure with preserved ejection fraction (HFpEF) patients, who account for 32.2% of the cohort, essentially represent a highly heterogeneous syndrome whose pathological basis may encompass multiple phenotypes such as hypertensive heart disease, coronary artery disease, and obesity/metabolic cardiomyopathy; this etiological diversity may lead to differences in symptom-driven mechanisms, yet the current network model has not conducted subgroup stratification analysis. Meanwhile, the symptom scales used in this study have only undergone Chinese localized validation, so there may be barriers to cross-cultural applicability when extrapolating the study conclusions to non-Chinese cultural regions, and cautious interpretation is required. Although the utilization rate of beta-blockers (91.8%) was recorded, specific dosage data and target achievement status were not systematically collected; given that lipophilic drugs (e.g., metoprolol) may affect anxiety manifestations through central penetration, insufficient dosage may result in residual symptoms or changes in symptom expression patterns,^[32]^ which poses a potential confounding factor for interpreting “anxiety” as a core symptom, and future studies need to integrate drug concentration monitoring to deepen the analysis of “dose-symptom” correlations. Additionally, the single-center sample may limit the generalizability of the conclusions, necessitating multi-center validation in the East China region; furthermore, the cross-sectional design fails to capture the dynamic evolution of the symptom network, so it is recommended that future research integrate wearable devices to achieve real-time biosensing and dynamic modeling. Symptom network analysis is also vulnerable to interference from unmeasured confounders (e.g., subclinical inflammation, autonomic dysfunction): for example, fluid retention dominated by renin-angiotensin-aldosterone system activation in the heart failure with reduced ejection fraction (HFrEF) group may strengthen the “edema-weight gain” association, while chronic microinflammation in the HFpEF group may simultaneously exacerbate fatigue, anxiety, and sleep disorders, leading to spurious correlations.^[33]^

## 5. Conclusion

Based on the systematic observation of 550 patients with CHF during CR, this study constructed a symptom network model for the first time. It revealed a 3-dimensional hub structure with “fatigue, exertional shortness of breath, and anxiety” at its core. Five symptom clusters were extracted through exploratory factor analysis, with the fatigue-nutritional imbalance group occupying a central position. Symptom network analysis indicated that fatigue was the most influential symptom. In light of these findings, a hierarchical intervention is recommended: resistance training combined with branched-chain amino acid supplementation for fatigue; threshold breathing training for shortness of breath after exercise; and cognitive behavioral therapy for anxiety to reduce symptom burden and promote the implementation of CR.

## Acknowledgments

We thank all the medical staff in the Department of Cardiology of the Sixth People’s Hospital of Nantong.

## Author contributions

**Conceptualization:** Fengfei Zhou, Bailing Zhang, Yi Lu, Qingqing Yang.

**Data curation:** Fengfei Zhou, Yi Lu, Qingqing Yang.

**Formal analysis:** Yi Lu, Qingqing Yang.

**Funding acquisition:** Bailing Zhang, Qingqing Yang.

**Investigation:** Bailing Zhang.

**Methodology:** Bailing Zhang.

**Project administration:** Bailing Zhang.

**Supervision:** Fengfei Zhou.

**Validation:** Fengfei Zhou.

**Writing – original draft:** Fengfei Zhou, Qingqing Yang.

**Writing – review & editing:** Fengfei Zhou, Qingqing Yang.
